# Comparison of a new optical biometer and a standard biometer in cataract patients

**DOI:** 10.1186/s40662-016-0059-1

**Published:** 2016-10-17

**Authors:** Pipat Kongsap

**Affiliations:** Department of Ophthalmology, Prapokklao Hospital, Chanthaburi, 22000 Thailand

**Keywords:** Intraocular lens power calculation, Partial coherence interferometry, Optical low coherence reflectometer, Optical biometer, Cataract surgery

## Abstract

**Background:**

Cataract surgery is the most common surgical procedure in ophthalmology. Biometry data and accurate intraocular lens (IOL) calculations are very important in achieving the desired refractive outcomes. The aim of this study was to compare measurements using a new optical low coherence reflectometry (OLCR) biometer (OA-2000) and the gold standard partial coherence interferometry (PCI) optical biometer (IOLMaster 500).

**Methods:**

Ocular biometry of cataract patients were measured by the OA-2000 and IOLMaster 500 to compare keratometry (K), axial length (AL), anterior chamber depth (ACD), white-to-white (WTW) diameter, and IOL power using the SRK/T formula.

**Results:**

One hundred and two eyes of 68 cataract patients were evaluated with the two optical biometers. The mean values of the AL, K, ACD, and WTW differed very little (OCLR biometer, 23.12 mm, 44.50 diopters (D), 3.06, and 11.64 mm, respectively; PCI biometer, 23.18 mm, 44.6 D, 3.15, and 11.86 mm, respectively), but the differences were significant (all, *p* ≤ 0.05). The AL, K, and ACD showed excellent correlations (*r* = 0.999, 0.980, and 0.824, respectively; all *p* < 0.001); however, there was a weak correlation of the WTW diameter between the two devices (*r* = 0.256). The IOL powers using the SRK-T formula derived from both instruments were very similar, with an excellent correlation (*r* = 0.989). The mean difference between the two instruments was 0.32 D.

**Conclusions:**

The OLCR biometer showed very a strong agreement with the standard PCI optical biometer for almost all ocular biometry measurements, except for the WTW diameter.

**Trial registration:**

TCTR20160614003; date 06/09/2016; ‘retrospectively registered’.

## Background

Cataract surgery is the most common ophthalmic surgical procedure, but accurate intraocular lens (IOL) calculations are very important in achieving the desired refractive outcomes. Biometry data, including the axial length (AL), keratometry (K), and anterior chamber depth (ACD), are necessary for an accurate power. Historically, the AL was measured using A-scan ultrasound biometry, and a previous study of ultrasound (US) biometry [1] reported that 54 % of errors in the predicted refraction after IOL implantation could be attributed to errors in AL measurements.

The IOLMaster 500 (Carl Zeiss Meditec, Dublin, CA, USA), an optical biometry device for AL measurements, has been shown to possess higher precision and greater reproducibility than US biometry [2–4]. It uses the principle of partial coherence interferometry (PCI) to obtain the AL, and also measures the K value, ACD, and white-to-white (WTW) diameter. The advantages of optical biometry compared with applanation US measurements include a lower risk for trauma and infection, increased patient comfort, and improved accuracy and repeatability of measurements [5]. The IOLMaster 500 is currently considered the gold standard for AL measurements [6–8].

The IOLMaster 500 optical biometer measures K values using six spots of light projected onto the cornea in a 2.5 mm zone. The AL is measured using the PCI method, and the ACD is measured using lateral slit illumination. Consistent with the manufacturer’s recommendations, five AL and ACD measurements and three keratometry measurements were performed with the PCI biometer.

The OA-2000 (Tomey, Nagoya, Japan) is the newest instrument used for optical biometry. It measures ocular biometry by using the principle of low coherence reflectometry (OLCR). This instrument measures the K value, AL, ACD, WTW diameter, lens thickness, pupil size, and central corneal thickness (CCT). Although the instrument is fast and easy-to-use, only one study has evaluated its accuracy.

The OA-2000 optical biometer measures corneal curvature using a placido disc-based topography technique with nine rings, each with 256 points, in a 5.5 mm zone projected onto the cornea. The Fourier domain method uses high-speed tissue penetration and is equipped with an automatic search function for CCT, ACD, lens thickness (LT), pupil diameter, WTW diameter, and AL measurements. A search function automatically detects a measurable point, even if the lens is not transparent. The instrument can perform ten consecutive scans per measurement without the need for realignment.

The objective of this study was therefore to compare measurements of the AL, K, ACD, and WTW diameter using the new OA-2000 optical biometer and the gold standard IOLMaster 500 optical biometer.

## Methods

Cataract patients examined between June 2015 and July 2015 at the Prapokklao Hospital Eye Clinic, Chanthaburi, Thailand were included in this study. The study adhered to the tenets of the Declaration of Helsinki and was approved by the Prapokklao Hospital Ethical Board Committee, and patient informed consent was obtained before each study. Eyes with previous ocular surgery and ocular diseases such as glaucoma, retinal disorder, and corneal diseases were excluded.

Measurements of ocular biometry were performed with the new OLCR biometer and the PCI biometer by two experienced examiners. The AL, ACD, WTW diameter, and keratometry were analyzed and compared between the two instruments. For AL measurements, both devices were set to the same immersion biometry algorithm. The averages of both flat and steep corneal curvatures were reported in diopters (D), and the WTW diameter values were reported in mm. Statistical analyses involving the mean, standard deviation, and minimum/maximum values were performed using SPSS statistical software for Windows, version 19.0 (SPSS, Chicago, IL, USA). Pearson’s correlation and intraclass correlation coefficients were used to determine relationships between the groups. Data between the instruments were compared using the Student’s *t-*test, and Bland-Altman graphs were used to show measurement differences of mean values. Bland-Altman graphs were also used to assess the agreement of measurements between the two devices. A value of *p* < 0.05 was considered statistically significant.

## Results

One hundred and two eyes of 68 patients (32 males and 36 females), with a mean age of 55.81 ± 11.51 years [± standard deviation (SD); range, 44-85 years] were recruited for this study. Fourteen eyes with dense nuclear cataracts could not be measured using the PCI biometer, but 13 eyes of these eyes could be measured using the new biometer. The 14 eyes with missing AL measurements were excluded from the study. Table 1 shows the values of the four ocular parameters (AL, K, ACD, and WTW diameter) and IOL power obtained from the new biometer and the reference biometer using the SRK/T formula. The mean values of all measurements, except the WTW distance and the calculated IOL power, showed excellent correlations between the two biometers. On average, AL, ACD, and K measurements with the new OCLR biometer showed smaller values compared with the PCI biometer. All differences were statistically significant (all, *p* ≤ .001). The mean difference and the SD of the AL, K, and ACD measurements were small, and the highest value was the WTW diameter. The new OCLR biometer also showed lower mean values than the PCI biometer for the WTW diameter, and the amplitude of the difference was greater than the AL, K, and ACD values.

**Table 1:** The differences in parameters between the new OCLR and the standard PCI biometers

Parameter	New biometer	PCI biometer	Difference	*p*-Value	*r*-Value	ICC
Axial length (mm)						
Mean ± (SD)	23.12 ± 1.34	23.18 ± 1.08	-0.05 ± 0.05	<0.001	0.999	0.996
Range	20.79, 26.5	20.93, 26.60	-0.16, 0.05^a^			
Keratometry (D)						
Mean ± (SD)	44.50 ± 1.8	44.60 ± 1.81	-0.11 ± 0.37	<0.001	0.980	0.980
Range	40.44, 47.38	40.57, 47.37	-0.84, 0.63			
ACD (mm)						
Mean ± (SD)	3.06 ± 0.39	3.15 ± 0.39	-0.09 ± 0.24	<0.001	0.824	0.824
Range	2.27, 3.10	2.29, 3.16	-0.58, 0.39			
WTW (mm)						
Mean ± (SD)	11.64 ± 0.77	11.86 ± 0.48	-0.21 ± 0.82	0.018	0.259	0.231
Range	8.74, 13.83	10.84, 12.80	-1.85, 1.42			
IOL power (D)						
SRK/T						
Mean ± (SD)	20.79 ± 2.81	20.44 ± 2.63	0.32 ± 0.60	<0.001	0.989	0.988
Range	12.5, 27.5	12.0, 27.0	-1.53, 0.88			

*OCLR*= optical low coherence reflectometry, *PCI*= partial coherence interferometry, *SD*= standard deviation, *D*= diopters, *ACD*= anterior chamber depth, *WTW*= white-to-white corneal diameter, *IOL*= intraocular lens, *r value*= Pearson’s correlation coefficient, *ICC*= intraclass correlation coefficient

^a^95 % limits of agreement

**Fig. 1:**
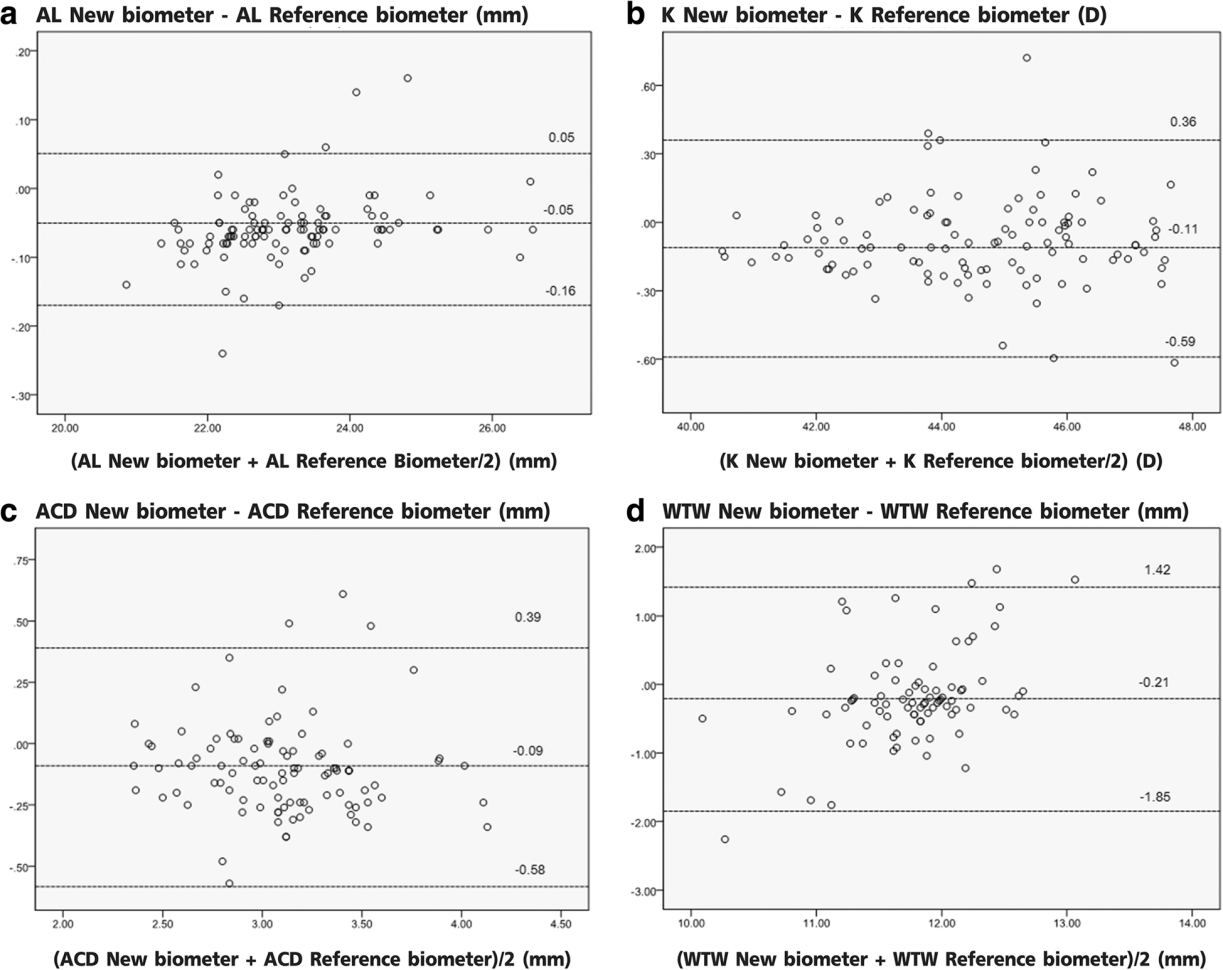
A Bland-Altman plot comparing parameter measurements between the new optical low coherence reflectometry biometer and the partial coherence interferometry biometer. **a** axial length; **b** keratometry; **c** anterior chamber depth; and **d** white-to-white corneal diameter

Correlations of ocular parameters derived from both biometers were excellent except for the WTW diameter (Table 1). The correlations were very strong for AL (*r* = 0.999), K (*r* = 0.980), and ACD (*r* = 0.824), but weak for the WTW diameter (*r* = 0.259). Figure 1a-d show these differences using Bland-Altman graphs for the AL, K, ACD, and WTW diameter. The Bland-Altman analyses also showed good agreement between the two operators for ocular parameter measurements, except that the WTW diameter values in cataract patients had wider 95 % limits of agreement (range, -1.85-1.42 mm). A few measurements were outliers, but in most cases, the differences between the two instruments were considered clinically insignificant for standard IOL calculations.

**Fig. 2:**
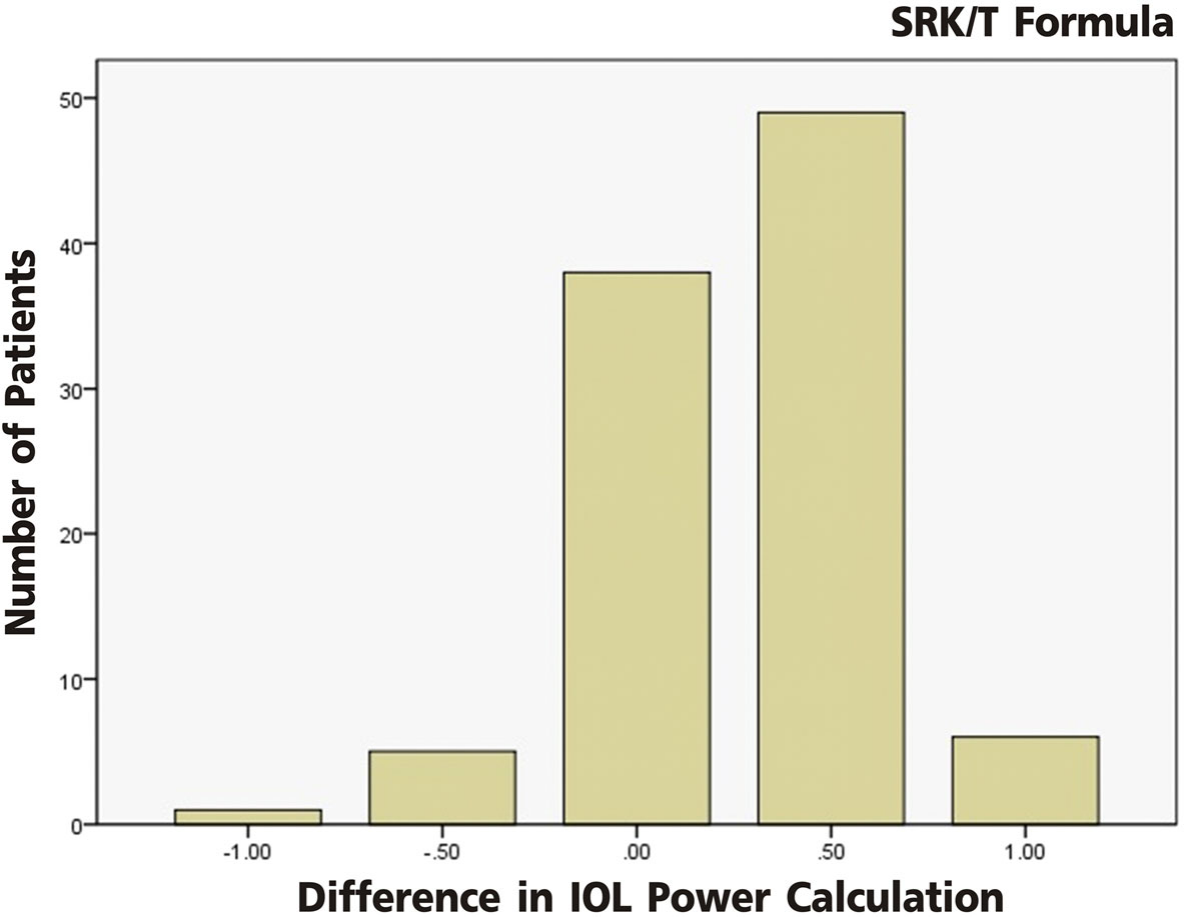
The distribution of the intraocular lens (IOL) power differences: new optical low coherence reflectometry biometer - the partial coherence interferometry biometer = the IOL power difference

For IOL power calculations, the mean difference between the two instruments was 0.32 D. In 93 % of the eyes, the mean IOL power difference between the two devices was ≤ 0.50 D (Fig. 2).

## Discussion

Accurate biometric data are essential for achieving good surgical outcomes and patient satisfaction after cataract and refractive surgery. Currently, several instruments are available, including optical coherence tomography, PCI, and OLCR. AL measurements using PCI of the IOLMaster 500 is considered the gold standard and is comparable to other instruments for routine use. This biometer also measures the K, ACD, and WTW diameter in an automated mode that allows greater patient comfort. The PCI biometer has been reported to have good repeatability and accuracy for AL measurements, and many studies have reported the accuracy of the PCI biometer when compared with traditional ultrasound biometry [9, 10]. However, realignment before measurements of each parameter is necessary when using the PCI biometer.

A non-contact imaging instrument using OLCR (Lenstar), has been compared with the IOLMaster 500 PCI biometer. Hoffer [11] reported excellent correlations between AL, ACD, and K measurements in cataract patients. The new OLCR biometer (OA-2000) is a recently developed biometer that can measure the ACD, CCT, LT, and AL. The preceding model (OA-1000) was incapable of acquiring keratometry measurements, but the newer OA-2000 can perform these measurements. The OA-1000 showed good reproducibility in measurements of the AL and ACD, and when compared with the PCI biometer [10, 12]. Furthermore, the Lenstar and OA-2000 instruments automatically performed the measurements without the need for realignment.

In the present study, we compared the AL, ACD, K, and WTW diameter values of two optical biometers (the new OCLR biometer and the PCI biometer) in cataract patients. Correlations of AL, ACD, and K values were excellent, except for the WTW diameter. The correlations were very strong for AL (*r* = 0.999), K (*r* = 0.980), and ACD (*r* = 0.824), but weak for the WTW diameter (*r* = 0.259).

Excellent correlations between the two devices were found for AL measurements. The mean AL using the new OCLR biometer was smaller than that using the PCI biometer (average, 0.05 mm). The difference between the QA-2000 and IOLMaster 500 was small, but still statistically significant. The differences between the new OCLR biometer and the PCI biometer or standard OCLR biometer (Lenstar) reported in a previous study [13] showed similar results (averages, 0.05 and 0.03 mm, respectively), although these differences may not be clinically relevant.

Keratometry measurements using the new OCLR biometer with a 5.5 mm zone placido device differed from measurements using the PCI biometer with 2.3 mm high density autokeratometry, but the correlation between the instruments was very strong (*r* = 0.98). The mean K value using the new OCLR biometer was lower than that using the PCI biometer (0.11 D). Goebels [13] found a high correlation between the new OCLR biometer and the PCI biometer (*r* = 0.83), and higher correlations between the standard OCLR biometer (Lenstar) and the PCI biometer (*r* = 0.93). The higher correlation between the standard OCLR biometer (Lenstar) and the PCI biometer may result from the similar measurement techniques used by these instruments.

The new OCLR biometer uses OLCR whereas the PCI biometer uses lateral slit illumination for ACD measurements. Excellent correlation was found between the new OCLR biometer and the PCI biometer (*r* = 0.82). The difference of ACD values between the two devices was approximately 0.09 mm in the present study, but the difference was 0.18 mm in a previous study [13]. Limampa [14] reported a difference of 0.2 mm in ACD measurements using a standard OCLR biometer (Lenstar) in comparison with a PCI biometer. Goebels [13] reported a difference of 0.08 mm in ACD measurements using a standard OCLR biometer (Lenstar) in comparison with a new OCLR biometer.

There was a weak correlation between the new OCLR biometer and the PCI biometer (*r* = 0.26) for WTW diameter measurements in the present study. The mean difference measured by the two instruments was 0.21 mm, but a higher SD was found in the new OCLR biometer (SD = 0.77). WTW diameter measurements using the IOLMaster 500 have been reported to be very accurate [15]. However, there has been no study of WTW diameter measurements using the new OCLR biometer (OA-2000). Like the AL-scan biometer, Srivannaboon [16] reported that the weak correlations could result from a difference in the algorithms for edge detection around the iris and the dissimilarity of the light source for image acquisition between the devices. An infrared light source (used in the IOLMaster 500) has been used in ophthalmic devices for eye tracking with high accuracy for many years, thus it might be a better choice for WTW diameter measurements.

The IOL powers using the SRK-T formula derived from both devices were quite similar, with an excellent correlation (*r* = 0.989). The mean difference between the two devices was 0.32 D, but it was less than the increment of the IOL power step of 0.5 D. In 93 % of eyes, the mean IOL power difference between the two devices was ≤ 0.50 D. These small differences are not clinically significant for most cataract patients. A clinically significant impact on IOL power should be considered if the WTW diameter measurement is required in the Holladay 2 formula. The IOL power between the two devices may therefore be different.

The penetration of the new OCLR biometer is better than the PCI biometer for the measurement of biometric data in eyes with a dense cataract. In the present study, the AL of 14 eyes with dense cataracts could not be measured with the PCI biometer, but 13 eyes of these eyes could be measured using the new OCLR biometer.

## Conclusion

The OLCR biometer showed very strong agreement with the standard optical biometer for almost all ocular biometry measurements. Correlations with the IOLMaster 500 were excellent except for the WTW corneal diameter. However, there were no data involving intraoperator repeatability in this study, which should be included in future studies, together with verification of the WTW diameter measurements.
